# Topical *Gynura procumbens* as a Novel Therapeutic Improves Wound Healing in Diabetic Mice

**DOI:** 10.3390/plants10061122

**Published:** 2021-06-01

**Authors:** Nutda Sutthammikorn, Volaluck Supajatura, Hainan Yue, Miho Takahashi, Sunee Chansakaow, Nobuhiro Nakano, Pu Song, Takasuke Ogawa, Shigaku Ikeda, Ko Okumura, Hideoki Ogawa, François Niyonsaba

**Affiliations:** 1 Department of Microbiology, Faculty of Medicine, Chiang Mai University, Chiang Mai 50200, Thailand; nutda@varee.ac.th (N.S.); volaluck.supajatura@gmail.com (V.S.); 2 Atopy (Allergy) Research Center, Juntendo University Graduate School of Medicine, Tokyo 113-8421, Japan; h-yue@juntendo.ac.jp (H.Y.); m.takahashi@juntendo.ac.jp (M.T.); nbnakano@juntendo.ac.jp (N.N.); songpu@fmmu.edu.cn (P.S.); ikeda@juntendo.ac.jp (S.I.); kokumura@juntendo.ac.jp (K.O.); ogawa@juntendo.ac.jp (H.O.); 3 Department of Dermatology and Allergology, Juntendo University Graduate School of Medicine, Tokyo 113-8421, Japan; t-ogawa@juntendo.ac.jp; 4 Department of Pharmaceutical Sciences, Faculty of Pharmacy, Chiang Mai University, Chiang Mai 50200, Thailand; chsunee@gmail.com; 5 Department of Dermatology, Xijing Hospital, Fourth Military Medical University, Xi’an 710032, China; 6 Faculty of International Liberal Arts, Juntendo University, Tokyo 113-8421, Japan

**Keywords:** angiogenesis, *G. procumbens*, skin, traditional medicine, wound healing

## Abstract

Nonhealing wounds are major socioeconomic challenges to healthcare systems worldwide. Therefore, there is a substantially unmet need to develop new drugs for wound healing. *Gynura procumbens*, a herb found in Southeast Asia, may be an effective therapeutic for nonhealing diabetic wounds. The aim of this study was to evaluate the efficacy of *G. procumbens* on wound healing in the diabetic milieu. *G. procumbens* extract was obtained using 95% ethanol and its components were determined by thin layer chromatography. Diabetes was induced in mice using streptozotocin. We found that *G. procumbens* extract contained stigmasterol, kaempferol and quercetin compounds. Topical application of *G. procumbens* on the wounded skin of diabetic mice accelerated wound healing and induced the expression of angiogenin, epidermal growth factor, fibroblast growth factor, transforming growth factor and vascular endothelial growth factor. Furthermore, *G. procumbens* promoted in vitro wound healing and enhanced the migration and/or proliferation of human endothelial cells, fibroblasts, keratinocytes and mast cells cultured in diabetic conditions. Finally, *G. procumbens* promoted vascular formation in the diabetic mice. To the best of our knowledge, this is the first study that evaluates in vivo wound healing activities of *G. procumbens* and activation of cells involved in wound healing process in diabetic conditions. The findings that *G. procumbens* accelerates wound healing and activates cells involved in the wound healing process suggest that *G. procumbens* might be an effective alternative therapeutic option for nonhealing diabetic wounds.

## 1. Introduction

Nonhealing chronic wounds do not progress through the healing process in a timely manner and have become a major socioeconomic challenge to healthcare systems worldwide. To date, the prevalence rate for chronic nonhealing wounds in industrialized countries is approximately 2% of the general population, similar to the prevalence rate for heart failure [1]. Chronic wounds include but are not limited to diabetic foot ulcers, venous leg ulcers, and pressure ulcers [2,3]. Although various therapeutic strategies have been pro-posed to treat chronic nonhealing wounds, including debridement, offloading, endovascular treatment, surgery to promote revascularization and the use of growth factors to promote wound healing, these wounds heal slowly and can worsen rapidly [4,5]. A recent study reported that chronic nonhealing wounds impact nearly 15% of Medicare beneficiaries (8.2 million), demonstrating the economic impact and burden of chronic nonhealing wounds in the Medicare population [1]. There is, therefore, a substantially unmet need to develop new interventions that are effective and inexpensive.

*Gynura procumbens* (Lour.) Merr. is a small plant approximately 1–3 m in height with a fleshy stem and ovate-elliptical or lanceolate shaped leaves. It is a perennial plant that belongs to the Asteraceae family and is commonly found in tropical Asian countries such as China, Thailand, Indonesia, Malaysia and Vietnam. Leaf extracts of *G. procumbens* are used in folk medicine to treat various ailments, such as fever, inflammation, migraines, rheumatism, cancer, viral infections and hypertension [6]. In addition, the extracts of *G. procumbens* leaves exhibit antihyperglycemic, antihyperlipidemic and hypoglycemic properties in diabetic rats [7] and promote wound healing in healthy rats [8]. The wound healing process consists of hemostasis, inflammation, proliferation and remodeling [9,10]. This process involves several cell types, including keratinocytes, fibroblasts, endothelial cells and mast cells [11]. Moreover, various growth factors, such as angiogenin (ANG), epidermal growth factor (EGF), fibroblast growth factor (FGF), transforming growth factor (TGF)-β, platelet-derived growth factor (PDGF) and vascular endothelial growth factor (VEGF), have been shown to facilitate the wound healing process [11].

Although *G. procumbens* has antiglycemic and wound healing properties, no report has been published on its effects on chronic nonhealing wounds or its ability to activate cells that play key roles in the wound healing process. Our main purposes were to evaluate the effects of an ethanolic extract of *G. procumbens* leaves on wound healing in normal and diabetic mice and to investigate its stimulatory properties in endothelial cells, fibro-blasts, keratinocytes and mast cells. We found that the *G. procumbens* extract efficiently accelerated wound healing and markedly promoted neovascularization in both healthy and diabetic mice. Moreover, the herb extract induced the expression of various growth factors, such as ANG, EGF, FGF, PDGF, TGF-β and VEGF, and enhanced the migration and/or proliferation of human endothelial cells, fibroblasts, keratinocytes and mast cells, which are vital prerequisites for the wound healing process. Considering the results, we propose that *G. procumbens* might be an alternative therapy for nonhealing chronic wounds, including diabetic wounds.

## 2. Results

### 2.1. Chemical Constituents of G. procumbens as Determined by TLC and Phytochemical Screening

**Figure 1:**
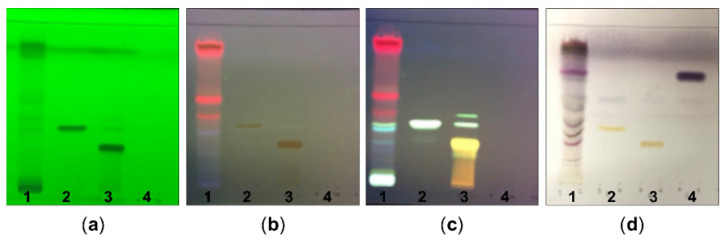
TLC chromatogram of ethanolic extract of *G. procumbens*: TLC profiles of ethanolic extract of *G. procumbens* (1), kaempferol (2), quercetin (3) and stigmasterol (4) observed under (**a**) UV at 254 nm, (**b**) UV at 366 nm, (**c**) natural product spraying reagent and (**d**) anisaldehyde-sulfuric acid spraying reagent.

We first examined the components of the ethanolic extract of *G. procumbens* as determined by TLC with detection under UV light at 254 nm (Figure 1a) and 366 nm (Figure 1b) and using a natural product spraying reagent (Figure 1c) and anisaldehyde-sulfuric acid spraying reagent (Figure 1d). The TLC chromatograms revealed kaempferol and quercetin (Figure 1c,d) and stigmasterol (Figure 1d) as chemical constituents of *G. procumbens* extract at Rf = 0.66, 0.38 and 0.32, respectively. The other standards, namely, chlorogenic acid, caffeic acid and rosmarinic acid, were not found in the composition of our ethanolic *G. procumbens* extract (data not shown). A previous study reported that chlorogenic acid is a component of an ethanolic extract of *G. procumbens* collected from HERBagus Sdn Bhd, Kepala Batas, Malaysia [12]. The discrepancy of this prior result and our study result may be due to differences in the geographical, climatic and/or experimental conditions. Moreover, a phytochemical screening of the ethanolic extract of *G. procumbens* revealed positive tests for phenolics, tannins, flavonoids, terpenes and other proteins (Table 1).

### 2.2. Ethanolic G. procumbens Extract Promotes Wound Healing in Both Normal and Diabetic Mice

To examine the effect of the ethanolic extract of *G. procumbens* on in vivo wound healing, full-thickness wounds were created on the dorsal back of mice and 0.5% *G. procumbens* was topically applied.

**Figure 2:**
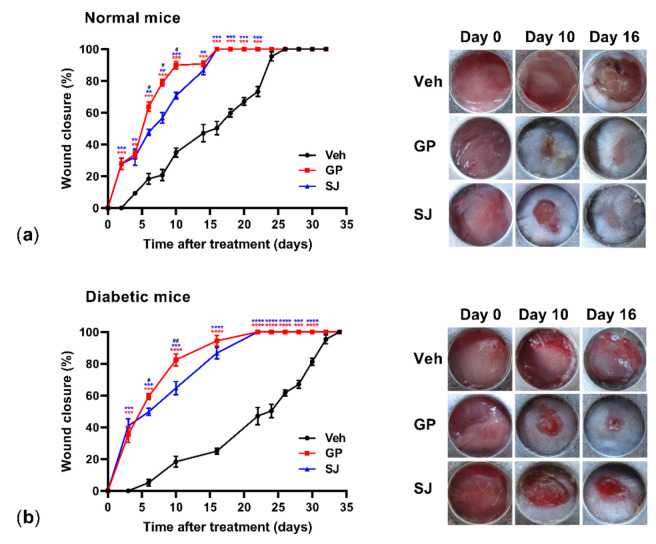
*G. procumbens* accelerates wound healing in the normal and diabetic mice: Dorsal full-thickness dermal wounds were created on (**a**) normal mice and (**b**) streptozotocin-induced diabetic mice and treated with Vaseline (Veh), 0.5% *G. procumbens* (GP) or 10% solcoseryl jelly (SJ) for up to 35 days. The left panels show the percentage of wound closure (n = 5 mice/group). Values represent the means ± SD, n = 5 mice/group. ** *p* < 0.01, *** *p* < 0.001 and **** *p* < 0.0001 for the comparisons between Vaseline (Veh: vehicle) and *G. procumbens* (GP, red) or solcoseryl jelly (SJ, blue). # *p* < 0.05 and ## *p* < 0.01 for the comparisons between *G. procumbens* (GP) and solcoseryl jelly (SJ). Right panels: representative photographs of wounds treated with Vaseline (Veh: vehicle), *G. procumbens* (GP) or solcoseryl jelly (SJ) on days 0, 10 and 16.

Solcoseryl jelly (10%) was used as a positive control for the treatment of diabetic wounds [13,14]. Compared to that of the vehicle-treated normal mice, significant wound healing activity was first observed on day 2 in animals treated with the 0.5% *G. procumbens* extract. The *G. procumbens*-treated wounds were completely healed by day 16, while the vehicle-treated mice were completely healed by day 26 (Figure 2a, left and right panels). On the diabetic mice, the *G. procumbens*-treated wounds started to significantly heal on day 2 and complete healing was observed on day 22, which differed from the vehicle-treated group, in which complete healing was observed on day 35 (Figure 1b, left panel). Overall, *G. procumbens* shortened the wound healing time in healthy and diabetic mice by 30% and 40%, respectively. In both the normal and diabetic groups, *G. procumbens* accelerated wound healing in the mice more rapidly than solcoseryl jelly. Compared to the effect of solcoseryl jelly, *G. procumbens* rapidly accelerated wound closure from day 6 to day 16 in both healthy and diabetic mice (Figure 2a,b, left panels). *G. procumbens* treatment caused neither toxicity nor mortality during or for at least 6 months after treatment (data not shown). All mice were healthy in growth, appearance and behavior.

### 2.3. G. procumbens Induces the Expression of Various Angiogenic Factors

Angiogenesis is critical for wound repair and is regulated by an extensive variety of angiogenic growth factors from various cells involved in the wound healing process.

**Figure 3:**
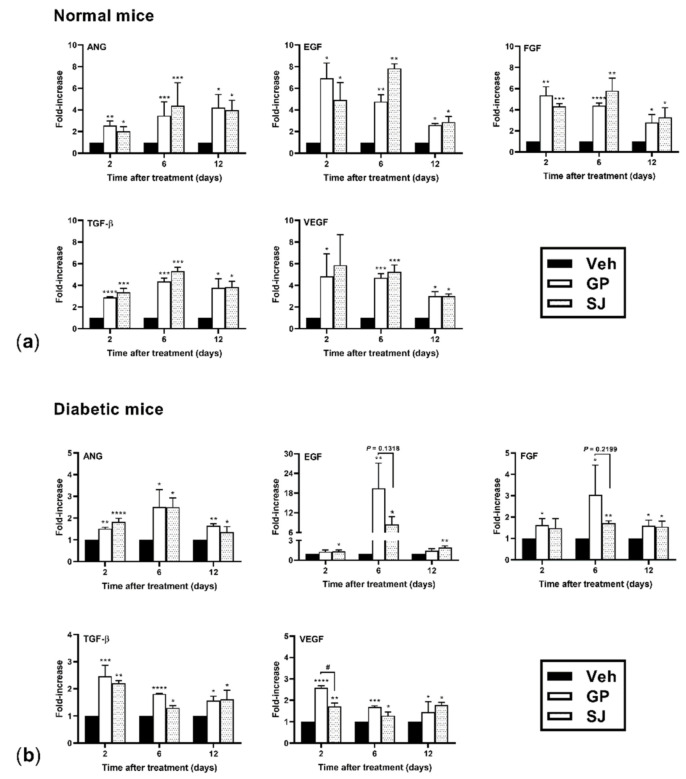
*G. procumbens* induces genes involved in wound healing in the normal and diabetic mice: The mRNA expression of ANG, EGF, FGF, TGF-β and VEGF in wounded skin from (**a**) normal mice and (**b**) diabetic mice treated with Vaseline (Veh: vehicle), 0.5% *G. procumbens* (GP) or 10% solcoseryl jelly (SJ) for 2–12 days (n = 5). * *p* < 0.05, ** *p* < 0.01, *** *p* < 0.001 and **** *p* < 0.0001 for the comparison between Vaseline (Veh: vehicle) and *G. procumbens* (GP) or solcoseryl jelly (SJ). # *p* < 0.05 for the comparison between *G. procumbens* (GP) and solcoseryl jelly (SJ).

Because *G. procumbens* induced wound healing, we speculated that it can also stimulate angiogenic growth factors. Skin tissues at the wound area were collected on days 2, 6 and 12 posttreatment and examined for the gene expression of angiogenic growth factors by RT-PCR. As shown in Figure 3a,b, *G. procumbens*-treated wound tissues of the normal control and diabetic mice displayed high expression of various angiogenic factors, including ANG, EGF, FGF, TGF-β and VEGF. No significant differences were found between *G. procumbens*- and solcoseryl-jelly-treated wounds with respect to the induction of angiogenic factors in the control mice. However, interestingly, *G. procumbens* markedly increased the expression of VEGF 2 days postinjury and tended to increase the levels of EGF (*p* = 0.1318) and FGF (*p* = 0.2199) 6 days postinjury in the diabetic mice compared with solcoseryl jelly (Figure 3b).

**Figure 4:**
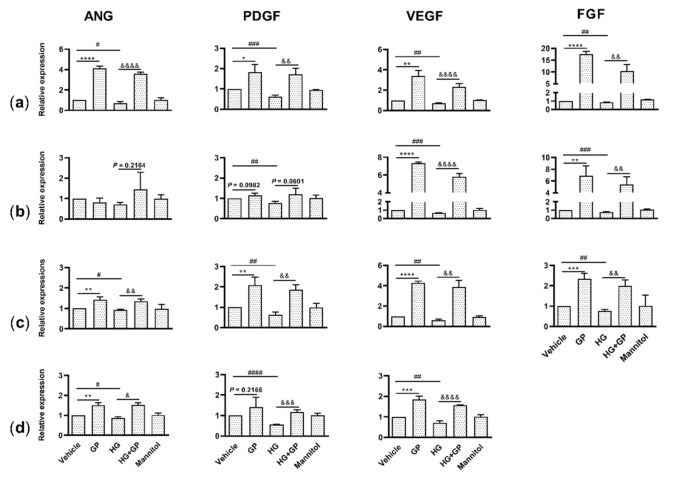
*G. procumbens* increases the expression of genes involved in wound healing in human keratinocytes, fibroblasts, endothelial cells and mast cells cultured in diabetic conditions: (**a**) Primary human keratinocytes, (**b**) fibroblasts, (**c**) endothelial cells and (**d**) the human mast cell line LAD2 were pretreated with 38 mM glucose (high glucose) or 38 mM mannitol for 24 h and then stimulated with 100 μg/mL *G. procumbens* (GP) or 0.1% DMSO in normal medium (Veh: vehicle) or 0.1% DMSO in high-glucose medium (HG) for 48 h. mRNA expression of ANG, PDGF, VEGF and FGF was measured with real-time PCR. The values are presented as the mean ± SD of five independent experiments. * *p* < 0.05, ** *p* < 0.01, *** *p* < 0.001 and **** *p* < 0.0001 for the comparisons between vehicle and *G. procumbens* (GP) in normal medium. & *p* < 0.05, && *p* < 0.01, &&& *p* < 0.001 and &&&& *p* < 0.0001 for the comparisons between vehicle (HG) and *G. procumbens* (HG+GP) in high-glucose medium. # *p* < 0.05, ## *p* < 0.01, ### *p* < 0.001 and #### *p* < 0.0001 for the comparisons between vehicle in normal medium and vehicle in high-glucose medium (HG).

The wound healing process involves the coordinated action of several types of cells, such as endothelial cells, fibroblasts, keratinocytes and mast cells, which produce a multitude of growth factors that are indispensable at each stage of the wound healing process [11]. To determine whether *G. procumbens* activated these cells to stimulate angiogenic factors under in vitro diabetic conditions, cells were treated with 38 mM glucose to mimic the diabetic milieu [15], and mannitol was used as an osmotic control for the high-glucose treatment [16]. We confirmed that glucose indeed attenuated the induction of angiogenic factors in keratinocytes, fibroblasts, endothelial cells and mast cells, but mannitol had no effect on cell activation (Figure 4a–d). Interestingly, *G. procumbens* significantly enhanced the mRNA expression of ANG, FGF, PDGF and VEGF in keratinocytes and endothelial cells under both normal and diabetic conditions (Figure 4a,c). The *G. procumbens* extract also markedly induced the expression of FGF and VEGF in fibroblasts (Figure 4b) and ANG and VEGF in mast cells (Figure 4d). The observation that *G. procumbens* stimulates angiogenic factors in human endothelial cells, fibroblasts, keratinocytes and human mast cells under diabetic conditions suggests that this herbal extract may have been involved in the angiogenesis of the diabetic mice.

### 2.4. G. procumbens Promotes Vascular Formation in Normal and Diabetic Mice

**Figure 5:**
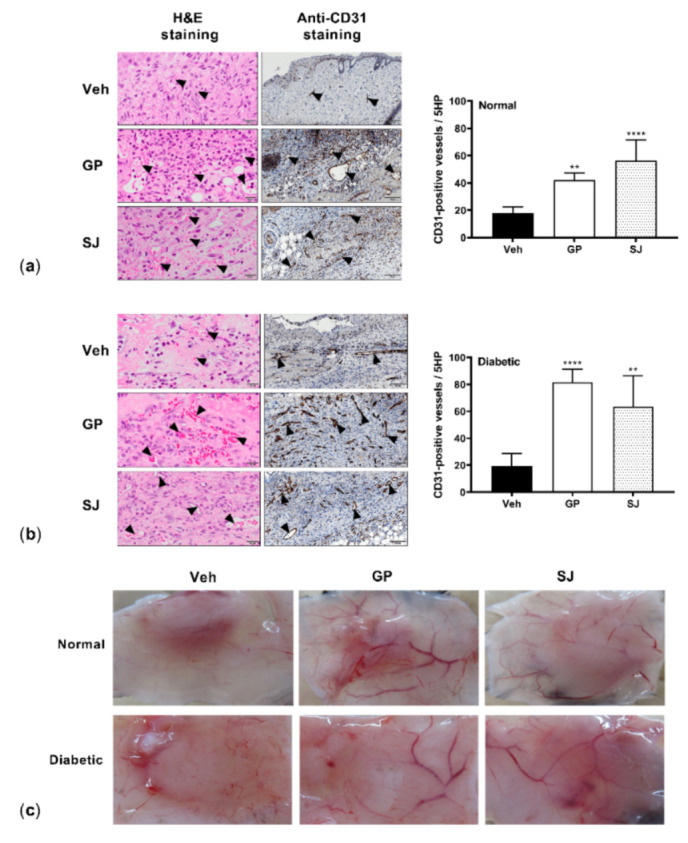
*G. procumbens* promotes blood vessel formation: Representative images of wounds from (**a**) normal and (**b**) diabetic mice treated with Vaseline (Veh: vehicle), 0.5% *G. procumbens* (GP) or 10% solcoseryl jelly (SJ). On day 8 post-treatment, sections were histologically stained with H&E (left panels) or immunohistochemically stained with anti-CD31 (middle panels). Scale bar = 20 μm for H&E and 100 μm for anti-CD31 staining. Right panels: the number of CD31-positive vessels. ** *p* < 0.01 and **** *p* < 0.0001 for the comparisons between the vehicle and *G. procumbens* (GP) or solcoseryl jelly (SJ). (**c**) Representative pictures of the macroscopic appearance of new blood vessels at the wound sites 8 days postinjury on the normal and diabetic mice treated with Vaseline (Veh: vehicle), 0.5% *G. procumbens* (GP) or 10% solcoseryl jelly (SJ).

Given that the *G. procumbens* extract increased the expression of angiogenic factors that are generally reduced in diabetic wounds [17], we hypothesized that this extract might encourage diabetic wound vascularity. Newly healed tissues collected on the 8th day of treatment were histologically examined. Hematoxylin and eosin staining showed an increased number of large vacuolar vessels in the *G. procumbens*-treated tissues from the normal mice (Figure 5a, left panels). This increased neovascularization was confirmed by an increased number of CD31-positive cells compared to the vehicle-treated wounds (Figure 5a, middle panels). Interestingly, in the diabetic mice, the *G. procumbens*-treated wounds had a strikingly increased number of vessels and displayed more CD31-positive cells than either the vehicle- or solcoseryl-jelly-treated wounds (Figure 5b, left and middle panels). A quantitative evaluation of the histological changes is shown in the right panels of Figure 5a,b.

Furthermore, macroscopic observations of repaired wounds on both the normal and diabetic mice clearly revealed vascular formation in the wound areas treated with *G. procumbens*. The vessels were remarkably increased in both number and size in the wounds treated with *G. procumbens* compared to the vehicle- and solcoseryl-jelly-treated wounds (Figure 5c).

### 2.5. G. procumbens Enhances Mast Cell Accumulation and Migration

**Figure 6:**
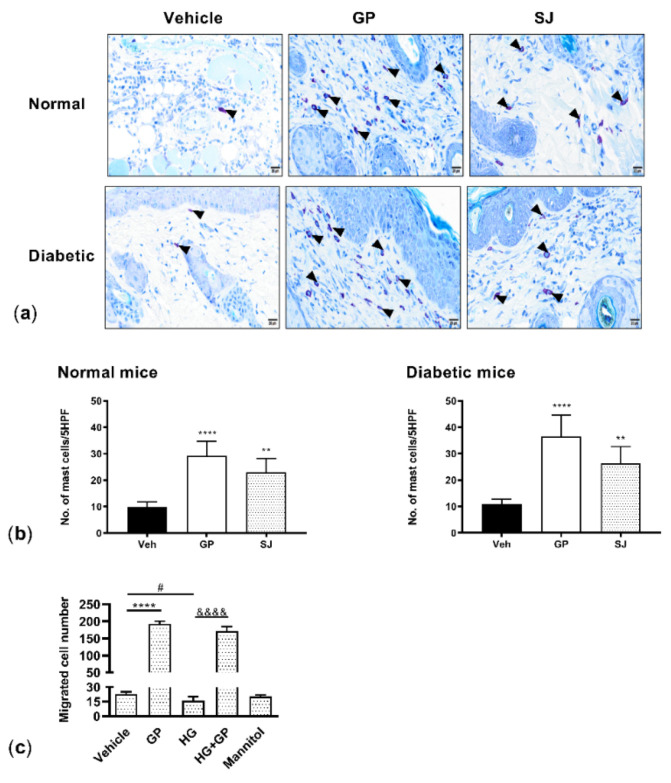
*G. procumbens* increases mast cell accumulation at wound sites and induces chemotaxis of LAD2 cells: Representative pictures of toluidine blue-stained wounds on (**a**) the normal and diabetic mice treated with Vaseline (Vehicle), 0.5% *G. procumbens* (GP) or 10% solcoseryl jelly (SJ) on day 8. n = 5. Arrows indicate the presence of mast cells. Scale bar = 20 μm. (**b**) Number of mast cells accumulated at the wound sites on normal and diabetic mice treated with Vaseline (Vehicle), 0.5% *G. procumbens* (GP) or 10% solcoseryl jelly (SJ) were quantified in 5 high-power fields (HPFs). (**c**) Mast cell chemotaxis: LAD2 cells pretreated with 38 mM glucose (high glucose) or 38 mM mannitol for 24 h and stimulated with 50 μg/mL *G. procumbens* (GP) or 0.1% DMSO in normal medium (vehicle) or 0.1% DMSO in high-glucose medium (HG) for 3 h. Migrated cells were counted under a light microscope. Bars represent the mean ± SD. ** *p* < 0.01 and **** *p* < 0.0001 for the comparison between vehicle and *G. procumbens* (GP) in the normal medium. &&&& *p* < 0.0001 for the comparison between the vehicle (HG) and *G. procumbens* (HG+GP) in high-glucose medium. # *p* < 0.05 between vehicle in normal medium and vehicle in high-glucose medium (HG). n = 3.

Mast cells accumulate in healing skin wounds and influence multiple phases of the wound healing process [18]. Tissues collected at wound areas on the 8th day of treatment were stained with toluidine blue and revealed that the *G. procumbens*-treated wound sites in the normal control mice displayed a substantial number of accumulated mast cells compared with the solcoseryl-jelly- and vehicle-treated wounds (Figure 6a, upper panels). *G. procumbens* treatment also remarkably increased the number of mast cells in the diabetic mice (Figure 6a, lower panels). The total number of accumulated mast cells in the healthy and diabetic mice was counted and is shown in Figure 6b.

The ability of *G. procumbens* to attract mast cells in the diabetic milieu was further confirmed by an in vitro chemotaxis assay using the LAD2 human mast cells cultured in high glucose. We observed that spontaneous mast cell migration was weakened under high-glucose conditions. Under both normal and diabetic conditions, *G. procumbens* dramatically induced mast cell migration. *G. procumbens*-induced cell migration resulted in an eightfold and tenfold increase under normal conditions and diabetic conditions, respectively (Figure 6c).

### 2.6. G. procumbens Promotes the Proliferation of Keratinocytes, Fibroblasts and Endothelial Cells

**Figure 7:**
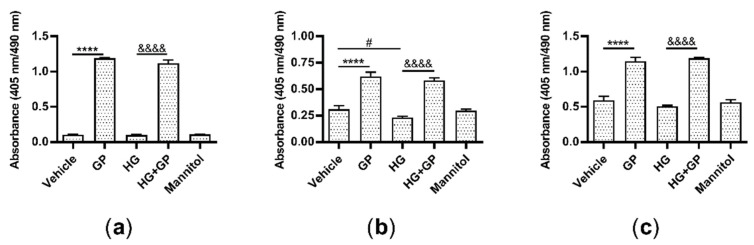
*G. procumbens* promotes the proliferation of human keratinocytes, fibroblasts and endothelial cells under diabetic conditions: (**a**) Primary human keratinocytes, (**b**) fibroblasts and (**c**) endothelial cells were pretreated with 38 mM glucose (high glucose) or 38 mM mannitol for 24 h and then stimulated with 50 μg/mL *G. procumbens* (GP) or 0.1% DMSO in normal medium (Veh: vehicle) or 0.1% DMSO in high-glucose medium (HG) for 48 h. Cell proliferation was assessed using a 5-bromo-2′-deoxyuridine (BrdU) Labeling and Detection Kit III. Values are presented as the mean ± SD of four independent experiments. **** *p* < 0.0001 for the comparison between vehicle and *G. procumbens* (GP) in normal medium. &&&& *p* < 0.0001 for the comparison between vehicle (HG) and *G. procumbens* (HG+GP) in high-glucose medium. # *p* < 0.05 for the comparison between vehicle in normal medium and vehicle in high-glucose medium (HG).

Wound healing is a dynamic reparative process that proceeds through a sequence of steps, including the proliferation and migration of different types of cells, such as keratinocytes, fibroblasts and endothelial cells [3]. First, we cultured these cells under high-glucose conditions and used a BrdU incorporation assay to determine whether *G. procumbens* can promote the cell proliferation. Significantly increased proliferation of keratinocytes, fibroblasts and endothelial cells was observed when these cells were treated with *G. procumbens* under both normal and diabetic conditions. A twelvefold increase was observed for keratinocytes under both normal and high-glucose conditions (Figure 7a), a twofold and threefold increase was observed for fibroblasts under normal and high-glucose conditions, respectively (Figure 7b), and a twofold increase was found in the endothelial cells under both normal and high-glucose conditions (Figure 7c).

### 2.7. G. procumbens Accelerates Wound Healing In Vitro

**Figure 8:**
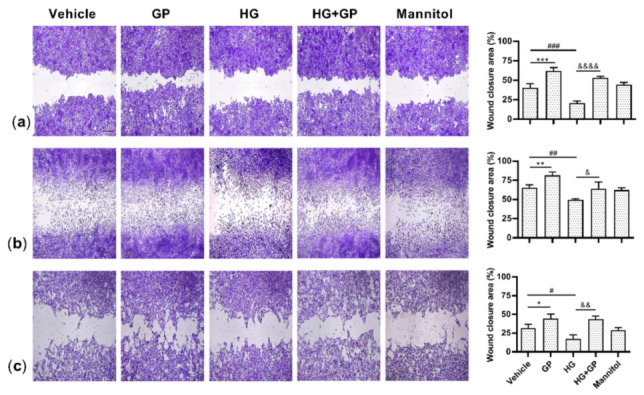
*G. procumbens* induces the migration of human keratinocytes, fibroblasts and endothelial cells under diabetic conditions. Cells were cultured in 38 mM glucose or 38 mM mannitol-containing medium for 24 h and then treated with 10 μg/mL mitomycin C for 2 h. A scratch assay was performed and the wounded monolayers were photographed under phase-contrast microscopy. Representative photographs of (**a**) primary human keratinocytes, (**b**) fibroblasts and (**c**) endothelial cells stimulated with 50 μg/mL *G. procumbens* (GP) or 0.1% DMSO in normal medium (vehicle) or 0.1% DMSO in high-glucose medium (HG) for 24 h. Right panels: the residual wound area was measured by ImageJ software. The values are presented as the means ± SD of four independent experiments. * *p* < 0.05, ** *p* < 0.01, *** *p* < 0.001 for the comparisons between vehicle and *G. procumbens* (GP) in normal medium. & *p* < 0.05, && *p* < 0.01 and &&&& *p* < 0.0001 for the comparison between vehicle (HG) and *G. procumbens* (HG+GP) in high-glucose medium. # *p* < 0.05, ## *p* < 0.01 and ### *p* < 0.001 for the comparisons between vehicle in normal medium and vehicle in high-glucose medium (HG).

Next, a scratch assay was used to assess the ability of *G. procumbens* to accelerate wound healing in vitro in keratinocytes, fibroblasts and endothelial cells that were cultured under both normal and high-glucose conditions. Because *G. procumbens* induces cell proliferation, all in vitro wound healing experiments were conducted in the presence of mitomycin C, an inhibitor of cell proliferation, to exclude the effect of proliferation on wound healing. Compared to the vehicle- or high-glucose-treated cells, the *G. procumbens*-treated cells rapidly migrated and covered the wound area under both normal and high-glucose conditions (Figure 8a–c). Quantification of the wound closure is shown in the right panels. *G. procumbens* promoted wound healing in the presence of mitomycin C, implying that *G. procumbens*-mediated wound healing can be mainly attributed to cell migration. The finding that *G. procumbens* induces cell proliferation and migration confirms the in vivo observation that *G. procumbens* promotes wound healing in diabetic subjects.

## 3. Discussion

Diabetic foot ulcers are common and costly to treat, leading to prolonged hospitalizations and approximately 25% of diabetic ulcerations resulting in lower extremity amputations [19,20].

Therefore, there is an urgent need for novel and relatively inexpensive approaches for the treatment of diabetic wounds. In this study, we report that an ethanolic extract of *G. procumbens* significantly accelerated wound healing, promoted vascularization in diabetic mice, increased the expression of angiogenic factors and induced the migration and/or proliferation of cells that contribute to wound healing in an in vitro diabetic milieu. To our knowledge, this is the first report showing that *G. procumbens* accelerates wound healing in diabetic subjects. We propose that *G. procumbens* is a therapeutic candidate for diabetic nonhealing wounds. The doses of *G. procumbens* (up to 200 μg/mL) used in this study were not cytotoxic, as assessed with a lactate dehydrogenase activity assay (data not shown). The safety of *G. procumbens* has been demonstrated in mice fed a high dose of *G. procumbens* (5000 mg/kg), which showed no toxicity even after long-term use [21]. 

*G. procumbens* has been reported to exhibit various biological activities, including but not limited to antihypertensive, cardioprotective, antihyperglycemic, fertility enhancement, anticancer, antimicrobial, antioxidant, organ protective, and anti-inflammatory activity, because of the presence of bioactive compounds [6]. Interestingly, the antihyperglycemic effect of *G. procumbens* has only been observed in diabetic animals, but not in normal animals, suggesting the specificity of the antidiabetic properties of *G. procumbens* [12]. Since the lifetime risk of a diabetic patient developing a foot ulcer is high, we aimed to investigate whether *G. procumbens* promotes wound healing activity in diabetic subjects. *G. procumbens* accelerated wound healing in diabetic mice and its effects were comparable to those of solcoseryl jelly, a commercially available hemodialysate known to be most effective when the wound healing process is impaired [13]. Solcoseryl promotes wound healing by stimulating wound contraction and the formation of granulation tissue, thereby reducing scar formation and promoting fibroblast migration and proliferation [13]. 

TLC studies demonstrated that our ethanolic extract of *G. procumbens* contained kaempferol, quercetin and stigmasterol, whereas phytochemical screening revealed the presence of phenolics, tannins, flavonoids and terpenes. In addition to promoting wound healing, these compounds have been demonstrated to display antioxidant properties. In fact, the antioxidant activity of *G. procumbens* is positively correlated with flavonoid contents [22]. Flavonoid compounds from ethanol extract of *G. procumbens* exhibit high antioxidant activity, as assessed using 2,2-diphenyl-1-picrylhydrazyl (DPPH) and 2,2-azino-bis-(3-ethylenebenzothiozoline-6-sulphonic acid) (ABTS) scavenging activity assays and ferric reducing antioxidant power (FRAP) assay [22], and inhibit the production of reactive oxygen species [23]. Kaempferol, a flavonoid present in different plants, possesses potent anti-inflammatory, antimicrobial and antioxidant activities [24,25]. Furthermore, kaempferol-treated diabetic and nondiabetic wounds show rapid re-epithelialization and tensile strength [26]. Kaempferol also induces keratinocyte migration [27] and acts on mast-cell-mediated wound healing [28]. Quercetin, another flavonoid found in the extract of *G. procumbens*, has antidiabetic, anti-inflammatory, antioxidant, antimicrobial and wound-healing effects, suggesting that quercetin is a promising drug for treating diabetes [29]. Moreover, Hujiahemaiti et al. reported that quercetin promotes the proliferation of human oral keratinocytes via upregulation of adhesion molecules and increases the re-epithelization rate of keratinocytes [30]. Stigmasterol, one of the most abundant plant sterols, displays various pharmacological and biological activities, such as antioxidant and anti-inflammatory activities [31], and stigmasterol ointment accelerates wound healing in animal models [32]. The ethanolic extract of *G. procumbens* also contained terpenes, which have a variety of properties, including antimicrobial, anti-inflammatory, antioxidant and wound healing activities [33,34]. The observation that wounds in normal and diabetic mice treated with the ethanolic extract of *G. procumbens* were not infected may be explained in part by the presence of flavonoids and tannins, which display antimicrobial activities [35]. Although further studies are needed to clarify which bioactive components of our herbal extract are more effective with respect to wound healing, it seems that the wound healing activities of *G. procumbens* are not the result of a single component but rather a combination of compounds that are involved in wound healing activities. 

The impact of different solvents on extraction yields, phytochemical constituents and biological activities such as antioxidant, antimicrobial and antiproliferative effects [36] or antidiabetic properties [12] of *G. procumbens* has been reported. For example, flavonoids such as myricetin, quercetin, kaempferol and apigenin were more highly detected in crude ethanolic extract than ethyl acetate or other fractions [22]. In addition, Afandi et al. reported that the content of flavonoid compounds in *G. procumbens* extracts was higher in ethanol extract followed by methanol extract, whereas aqueous extract yielded the lowest content. In contrast, the content of phenolic compounds was higher in methanol extract followed by ethanol and aqueous extract. Furthermore, while ethanol extract exhibited a strong inhibitory effect on CYP3A4 and CYP1A2 enzymes, methanol extract showed the most active free radical scavenging activity [37]. In addition, among ethanol, chloroform, ethyl acetate, methanol and n-butanol used for extraction of *G. procumbens*, methanol extract displayed the highest radical scavenging activity and antimicrobial activity, while ethyl acetate yielded the highest phenolic content and chloroform yielded the lowest content [38].

Normal cutaneous wound healing includes sequential and overlapping phases of hemostasis/coagulation, inflammation, proliferation and remodeling. Disruption or dysregulation of any step of the wound healing phases leads to nonhealing/chronic wounds such as diabetic foot ulcers, pressure ulcers and venous ulcers [2,3]. Angiogenesis is initiated immediately after tissue injury and is mediated throughout the wound healing process. However, angiogenesis is decreased in diabetic patients, leading to insufficient neovascularization and decreased angiogenic growth factors [39]. It has been shown that the levels of angiogenic factors such as VEGF, FGF, PDGF and TGF-β are decreased in diabetic wound animal models [40,41]. Interestingly, the topical application of FGF, VEGF and PDGF has been shown to accelerate wound closure in diabetic mice compared to normal mice [39,41]. This was also observed in humans, where the topical application of PDGF was superior to a placebo in promoting wound healing in patients with diabetic foot ulcers [42] and VEGF gene transfer increased the vascularity of leg ulcers [43]. In addition to accelerating diabetic wound healing, we found that *G. procumbens* promoted neovascularization and elevated the expression of ANG, EGF, FGF, PDGF, TGF-β and VEGF in the diabetic mice and/or in cells involved in wound healing that were cultured under high-glucose conditions. ANG regulates angiogenesis, cell migration, proliferation and adhesion; EGF promotes angiogenesis and the proliferation and migration of keratinocytes; FGF plays a major role in angiogenesis, granulation tissue formation and re-epithelialization; PDGF stimulates cell migration and proliferation; TGF-β promotes inflammation, angiogenesis, re-epithelialization and tissue regeneration and VEGF increases neovascularization, angiogenesis, granulation tissue deposition and epithelialization [3,44,45]. Intriguingly, *G. procumbens* seemed to be more effective than solcoseryl jelly in stimulating EGF, FGF and VEGF and increasing the number of vessels in the diabetic wounds but not in the normal wounds, suggesting that *G. procumbens* may be a potential candidate for the treatment of diabetic wounds.

Keratinocytes, fibroblasts, endothelial cells and mast cells promote angiogenesis during the wound healing process through the production of growth factors such as ANG, EGF, FGF, PDGF, TGF and VEGF [3,44,45,46,47,48]. Furthermore, the aforementioned cells interact with each other, proliferate and migrate toward wound sites [49]. In in vitro studies mimicking diabetic conditions, *G. procumbens* showed increased expression of angiogenic factors in these cells and promoted their migration and proliferation. Cell migration and proliferation are two indispensable prerequisites of skin wound healing and predominantly occur during the proliferation phase, which consists of re-epithelialization, angiogenesis and granulation tissue formation [50]. During re-epithelialization, keratinocytes proliferate and migrate from the wound edges in an attempt to close the wound. During angiogenesis, endothelial cells escape from existing blood vessels and proliferate and migrate to the source of the angiogenic stimulus to form new capillary-like tubes in granulation tissue, fibroblasts migrate to the provisional matrix to degrade it and proliferate to form granulation tissue [50]. Mast cells accumulate within 24 h of injury and can increase as high as fivefold at wound edges. Mast cells regulate angiogenesis and the proliferation of endothelial cells, fibroblasts and keratinocytes [51]. However, recent studies have shown that the genetic depletion of mast cells does not affect normal re-epithelialization, granulation tissue formation or scar formation, suggesting that the exact role of mast cells in wound healing has yet to be determined [52,53].

To the best of our knowledge, this is the first study showing that *G. procumbens* accelerates wound healing in diabetic mice and activates various cells involved in wound healing process in high-glucose conditions. Therefore, we propose *G. procumbens* herbal extract as a new, affordable, indigenous pharmaceutical for patients with nonhealing wounds. The fresh leaves of *G. procumbens* are commonly consumed raw or used for cooking in Southeast Asia and scientifically, this plant has been shown to be safe for consumption. *G. procumbens* is used systemically and/or topically for treatment of various ailments due to the presence of bioactive compounds such as flavonoids and glycosides in this plant [6]. Although *G. procumbens* has been widely used, the underlying mechanisms by which this plant functions and exact chemical constituents involved have yet to be elucidated for the development of standardized drugs from this plant. Furthermore, it would be interesting to develop green and sustainable extraction methods, without the use of harmful solvents in the future.

## 4. Materials and Methods

### 4.1. Extraction of G. procumbens

Fresh leaves of *G. procumbens*, also known as “Pae Tum Punk” in Thai, were purchased from a local market in Chiang Mai Province, Thailand. The authenticity of the plant was verified by Dr. Angkhana Inta from the Department of Biology, Faculty of Science, Chiang Mai University, Thailand (voucher number: 7175), and the specimen was deposited at the Queen Sirikit Botanic Garden Herbarium, Thailand. The leaves were washed and air-dried and then ground to a fine powder. *G. procumbens* extraction from 500 g of dried leaves was performed using 95% ethanol and the extract mixture was filtered through cotton wool, followed by Whatman No. 1 filter paper (Sigma-Aldrich, St Louis, MO, USA). *G. procumbens* extraction using 95% ethanol was conducted according to a previously published method with some modifications [22,23]. Ethanol solvent was used because it has been demonstrated to be more efficient than other solvents such as methanol and aqueous solvents [37]. The filtrate was concentrated at 70 °C using a rotary evaporator (LTE Scientific, Oldham, UK) to obtain a brownish syrupy mass that was 2.7% of its original volume, which was then mixed with Vaseline to produce a 0.5% *G. procumbens* crude extract. The extract was maintained below 30 °C and used for animal wound healing studies. For the *G. procumbens* samples used in the in vitro experiments, the filtrate of the extract was concentrated at 40 °C, frozen at −40 °C and lyophilized to generate the semisolid form. The extract was dissolved in 50 mg/mL dimethyl sulfoxide (DMSO, Sigma-Aldrich), filtered through a 0.22-µm diameter membrane and stored at −30 °C until use. The final concentration of DMSO in each sample did not exceed 1% *v/v G. procumbens* extract for either animal or in vitro experiments. The samples were tested for bacterial and fungal contamination and the results showed that the *G. procumbens* extracts were sterile (data not shown).

### 4.2. Phytochemical Screening of the G. procumbens Extract

Thin layer chromatography (TLC) was used to investigate the chemical components of the *G. procumbens* ethanolic extract using the modified method of Abrika et al. [37]. Chloroform:methanol:formic acid (80:20:1) was used as the mobile phase and the reference standards were kaempferol, stigmasterol, quercetin, chlorogenic acid, caffeic acid and rosmarinic acid. Each sample was applied to a normal phase silica plate. After development in the chamber, the TLC plates were dried with a hairdryer and the components were detected under 254 nm and 365 nm ultraviolet (UV) light and the plate was sprayed with a freshly prepared anisaldehyde-sulfuric acid reagent. Rf values were used to compare the distances of the unknown spots and calculated as follows: Rf = migration distance of spot/migration distance of solvent. *G. procumbens* extract was subjected to various phytochemical tests to determine the chemical nature of the extract [54].

### 4.3. Animals

Male 8-week-old C57BL/6 mice were purchased from Japan SLC (Hamamatsu, Shizuoka, Japan) and maintained under specific pathogen-free conditions while consuming a standard diet and water ad libitum. Animals with unrelated health issues were excluded from the study. Animals were randomly allocated to normal and diabetic groups. The mice in the diabetic group were fasted for 6 h and then received a 0.2-mL intraperitoneal injection of streptozotocin (Sigma-Aldrich, 100 mg/kg body weight in citrate buffer at pH 4.5). One week after streptozotocin administration, the blood glucose levels were measured using an Accu-Chek glucose meter (Roche Diagnostics, Miami, FL, USA) and the mice with glycemia, glucose > 250 mg/dL, were considered diabetic [55]. The diabetic mice were monitored for polydipsia, polyuria and a high fasting blood glucose concentration. All procedures were approved by the Institutional Review Committee of Juntendo University and were conducted according to the National Institutes of Health Guide for the Care and Use of Laboratory Animals. The study was conducted according to the guidelines of the Declaration of Helsinki and approved by the Institutional Review Board of Juntendo University (protocol code 310070, approved on 21 February 2019).

### 4.4. In Vivo Wound Model and Treatment

The mice were anesthetized with 2.5% isoflurane. The dorsal portion of each mouse’s back was shaved and two 10 mm diameter full-thickness wounds were created using a circular biopsy punch under aseptic conditions. Incision of the muscle layer was carefully avoided and the skin tension remained constant during the procedure. Silicone splints were sutured to the wound perimeter to prevent wound contraction and the wound area was immediately measured using a Vernier caliper. Samples of 0.5% *G. procumbens* or Vaseline, used as the vehicle, were topically applied immediately after surgical excision and every 2 days until the wounds were completely healed. The wounds were covered with a hydrocolloid dressing (Tegaderm; 3 M Health Care, Tokyo, Japan) and cleaned with normal saline before treatment. The wounds were photographed and the wound areas were calculated using ImageJ software (NIH, Bethesda, MD, USA). The following formula modified from Cragg and Newman was used to calculate wound closure [56]:

% wound closure = (initial size − specific day size/initial wound size) × 100(1)

### 4.5. Cell Culture

Human umbilical vein endothelial cells and normal human epidermal keratinocytes were purchased from Kurabo Industries (Osaka, Japan), whereas normal human dermal fibroblasts were purchased from Lifeline Cell Technology (Osaka, Japan). Endothelial cells were cultured in HuMedia-EB2 supplemented with 2% fetal bovine serum, EGF (10 ng/mL), basic FGF (2 ng/mL), hydrocortisone (1.34 μg/mL), heparin (10 μg/ mL), gentamycin (50 μg/mL) and amphotericin B (50 ng/mL). Keratinocytes were routinely cultured in HuMedia-KG2 (Kurabo Industries) containing human EGF (0.1 ng/mL), insulin (10 μg/mL), hydrocortisone (0.5 μg/mL), gentamycin (50 μg/mL), amphotericin B (50 ng/mL) and bovine brain pituitary extract (0.4% vol/vol). Fibroblasts were grown in FibroLife serum-free medium (Lifeline Cell Technology) supplemented with l-glutamine (7.5 mM), basic FGF (5 ng/mL), insulin (5 μg/mL), ascorbic acid (50 μg/mL), hydrocortisone (1 μg/mL), gentamycin (30 μg/mL), amphotericin B (15 μg/mL) and 2% fetal bovine serum. Experiments were conducted using subconfluent cells (from 60% to 80% confluence) in the appropriate medium without supplements but with antibiotics [57]. LAD2 human mast cells (provided by Dr. A. Kirshenbaum at the National Institutes of Health, Bethesda, MD) were cultured in Stem Pro-34 medium containing nutrient supplements (Invitrogen, Carlsbad, CA, USA) supplemented with 2 mM l-glutamine (Invitrogen), 100 IU/mL penicillin and 100 μg/mL streptomycin (Meiji Seika, Tokyo, Japan) and 100 ng/mL human stem cell factor (Wako, Osaka, Japan) as previously reported [58].

### 4.6. Cell Migration

In vitro wound healing assay: Endothelial cells, fibroblasts or keratinocytes were cultured in normal medium or 38 mM glucose for 24 h. Following trypsinization, 0.5 × 10^5^ cells/well were seeded into collagen I-coated 96-well plates and cultured for 3 h at 37 °C. Cell monolayers were scratched using a 96-well WoundMaker (Essen BioScience, Ann Arbor, MI, USA), and the wells were washed with PBS to remove cellular debris. To exclude the influence of *G. procumbens*-induced proliferation on cell migration, the cells were pretreated with 10 μg/mL mitomycin C (Fujifilm, Tokyo, Japan) for 2 h before 24 h treatment of 50 μg/mL *G. procumbens*. Following treatment, adherent cells were stained with 0.5% crystal violet (Fujifilm), and images were recorded using a phase-contrast microscope (Keyence, Osaka, Japan). The wound closure area was measured with ImageJ software (National Institutes of Health, Bethesda, MD, USA).

The migration of the LAD2 cells was evaluated using a 48-well chemotaxis microchamber (Neuro Probe, Gaithersburg, MD, USA). Following treatment of the LAD2 cells with 38 mM glucose for 24 h, 27 μL of 50 μg/mL *G. procumbens* was added to the lower wells, and an 8-μm-pore polyvinylpyrrolidone-free polycarbonate membrane (Neuro Probe) precoated with 10 µg/mL human skin collagen type I for 2 h (Sigma-Aldrich) was placed between the lower and upper wells [58,59]. Fifty microliters of LAD2 cells (2 × 10^6^ cells/mL) were added to the upper chamber, followed by a 3 h incubation at 37 °C. After incubation, the membranes were stained with Diff-Quik, and the migrated cells were counted in 5 random high-power fields under a light microscope (Zeiss, Oberkochen, Germany). In preliminary experiments, the dose-dependent responses showed a bell-shaped curve with a peak at 50 µg/mL *G. procumbens*.

### 4.7. Cell Proliferation

Endothelial cells, fibroblasts and keratinocytes treated with 38 mM glucose for 24 h were trypsinized, and 1 × 10^4^ cells/well were cultured in collagen I-coated 96-well plates and stimulated with 50 µg/mL *G. procumbens* for 48 h at 37 °C. Cell proliferation was assessed using the 5-bromo-2′-deoxyuridine (BrdU) Labeling and Detection Kit III (Roche Diagnostics, Indianapolis, IN, USA) according to the manufacturer’s instructions. In brief, the cells were incubated with 10 µM BrdU for 4 h, and the cells that incorporated BrdU into DNA were detected using monoclonal anti-BrdU-peroxidase Fab fragments. The bound conjugate was visualized with the soluble chromogenic substrate ABTS (2,2′-azino-bis (3-ethylbenzothiazoline-6-sulphonic acid)) and measured using a microplate reader at a wavelength of 450 nm [60].

### 4.8. Total RNA Extraction and Real-Time Quantitative PCR

Total RNA from the skin tissue of normal and diabetic mice was extracted using an RNeasy Plus Universal Mini kit (Qiagen, Hilden, Germany), while RNA extraction from endothelial cells, fibroblasts, keratinocytes and LAD2 cells stimulated with 100 µg/mL *G. procumbens* for 48 h was performed using an RNeasy Plus Micro kit (Qiagen) according to the manufacturer’s instructions. First-strand cDNA was synthesized from 1 µg of total RNA using a ReverTra Ace qPCR RT kit (Toyobo, Osaka, Japan). Real-time PCR was performed with an Applied Biosystems StepOnePlus Real-time PCR system (Thermo Fisher Scientific, Waltham, MA, USA) by using TaqMan Universal PCR Master Mix and Assays-on-Demand primer/probe sets for ANG (Hs04195574_sH), EGF (Hs01099990_m1), FGF (Hs00266645_m1), TGF-β (Hs00998133_m1), VEGF (Hs00900055_m1) and PDGF (Hs00211916_m1). Each sample was analyzed in triplicate, the mRNA level measured in each sample was normalized to that of mRNA encoding an internal reference, β-actin, and the expression levels of individual genes were reported relative to their expression in the control samples.

### 4.9. Histologic and Immunohistochemical Analyses and Visualization of Vascular Formation at the Wound Site

The wound edge tissues were fixed in 10% neutral buffered formalin (Wako Pure Chemical Industries), processed, embedded in paraffin, sectioned in 3-µm slices and stained with hematoxylin and eosin. For mast cell detection, deparaffinized sections were stained with 0.05% toluidine blue at pH 2.5. Immunohistochemistry for angiogenesis was performed by staining tissues overnight with an anti-CD31 antibody (Abcam, Tokyo, Japan, ab28364), a marker of endothelial cells, at 4 °C. After washing with PBS, biotinylated goat antirabbit IgG was added and incubated at room temperature for 30 min. Diaminobenzidine tetrahydrochloride solution (DAKO, Tokyo, Japan) was then added, incubated at room temperature for 10 min and counterstained with hematoxylin. The number of blood vessels in 5 different high-power fields per section were counted. Images were acquired using the Olympus VS120 Virtual Slide Scanning System.

To visualize subcutaneous vascular formation at the wound site, a 1.5 cm × 1.8 cm full-thickness skin specimen from the newly repaired skin at the wound area was cut and washed 3 times with PBS. The skin specimen was placed upside down on a transparent dish and macroscopically visualized for vascular formation.

### 4.10. Statistical Analysis

Statistical analysis consisted of either ANOVA followed by the appropriate posthoc test or Student’s *t*-test using GraphPad Prism for Windows (Prism 8, GraphPad Software, San Diego, CA, USA). *p* < 0.05 was considered significant. The results are presented as the means ± SD.

## 5. Conclusions

The current study showed that an ethanolic extract of *G. procumbens* has the potential to accelerate the healing of diabetic wounds by inducing angiogenesis and promoting cell migration and proliferation. The safety of *G. procumbens* has been demonstrated in mice fed a high dose of *G. procumbens* (5000 mg/kg), which showed no toxicity even after long-term use [21]. Furthermore, fresh or cooked *G. procumbens* leaves are commonly consumed in Southeast Asia. Given the pharmacological activities, safety and low cost of *G. procumbens*, we propose *G. procumbens* herbal extract as a new, affordable, indigenous pharmaceutical for patients with nonhealing wounds. Further studies are needed to elucidate the mechanisms underlying the biological functions of *G. procumbens* for development of standardized drugs.

## 6. Patents

The patents resulting from the work reported in this manuscript fall under a domestic patent (Tokyo, Japan, reference number: 2018-214836).

## Figures and Tables

**Table 1:** Phytochemical screening of *Gynura procumbens* (Lour.) Merr. was determined by chemical reagents.

Test	Result
1. Alkaloid	
1.1. Dragendorff’s reagent	−
1.2. Wagner’s reagent	−
1.3. Marme’s reagent	−
1.4. Mayer’s reagent	−
2. Tannins (General test)	
2.1. Ferric chloride TS.	+
2.2. 0.5% Gelatin solution	+
2.3. 1% Lead acetate solution	−
2.4. 1% Quinine sulfate	−
3. Tannins (hydrolysable tannin)	
3.1. Ferric chloride TS.	−
3.2. Lime water	−
4. Tannins (condensed tannin)	
4.1. Vanillin reagent	−
4.2. Formalin-HCl reagent	−
5. Glycoside	
5.1. Antraquinone	−
5.2. Flavonoid	+
5.3. Saponin	−
5.4. Cyanogenic glycoside	−
5.5. Cardiac glycoside (steroid)	−
5.6. Cardiac glycoside (unsaturated lactone)	−
5.7. Cardiac glycoside (deoxy sugar)	−
6. Terpenoids	+
7. Protein	+
7.1. Xanthoproteic	+
7.2. Ninhydrin	+
7.3. Lead sulfide	+

Phytochemical tests were evaluated for their chemical group, e.g., flavonoids, alkaloids, glycosides, proteins, terpenoids using chemical reagents. (+) Positive result; (−) Negative result.

## Data Availability

The data presented in this study are available on request from the corresponding author.

## Abbreviations

ANG	Angiogenin
EGF	Epidermal growth factor
FGF	Fibroblast growth factor
*G. procumbens*	*Gynura procumbens*
PBS	Phosphate-buffered saline
PDGF	Platelet-derived growth factor
TGF	Transforming growth factor
VEGF	Vascular endothelial growth factor
